# The trafficking of women and girls in Taiwan: characteristics of victims, perpetrators, and forms of exploitation

**DOI:** 10.1186/s12905-017-0463-2

**Published:** 2017-11-09

**Authors:** Lanying Huang

**Affiliations:** Graduate School of Criminology, National Taipei University, 151 University Rd., San Shia District, New Taipei City, 23741 Taiwan

**Keywords:** Human trafficking, Women and girl victims, Exploitation, Immigrant workers

## Abstract

**Background:**

Prior to the passing of 2009 Human Trafficking Prevention Act (HTPA), human trafficking was underestimated in Taiwan. In the past, domestic trafficking in women and girls often targeted vulnerable groups such as young girls from poor families or minority groups. Since the 1990s, an increasing flow of immigrant women, mainly from Vietnam and Indonesia and some from China, into Taiwan has created a new group of Human Trafficking victims. The current study intends to identify, describe, and categorize reported and prosecuted human trafficking cases involving women and girls according to the HTPA in Taiwan.

**Methods:**

Using the court proceedings of prosecuted trafficking in women and girls cases under Taiwan’s HTPA from all 21 districts in Taiwan from 2009 to 2012 under the title keyword of ‘Human Trafficking’, this current study aims to categorize different patterns of existing trafficking in women and girls in Taiwan. The analysis is based on 37 court cases, involving 195 victimized women and girls and 118 perpetrators.

**Results:**

This study identifies six forms of Human Trafficking victims according to their country of origin, vulnerability status, and means of transport. This study found that women and girls suffer from both labor and sexual exploitation, from mainly domestic male perpetrators. While sexual exploitation is more evenly distributed among citizens and immigrants and affects both adults and minors, labor exploitation seems to be an exclusive phenomenon among women immigrant workers in the data.

**Conclusions:**

Human Trafficking cases in Taiwan share many of the similarities of Human Trafficking in other regions, which are highly associated with gender inequality and gender-based vulnerability.

**Electronic supplementary material:**

The online version of this article (10.1186/s12905-017-0463-2) contains supplementary material, which is available to authorized users.

## Background

### Trafficking in women and girls in Taiwan history

Human Trafficking (hereafter HT), either domestic or cross-national, is a gender issue [1]. Although the share of men among detected trafficking victims has been growing in the last decade, women and girls still make up more than 70% of the detected trafficking victims in the 2016 Global Report on Trafficking in Persons [2]. On the other hand, the extent of women’s rights and level of discrimination are listed as one of five dimensions in the Global Slavery Index, which aims to examine the population vulnerability within individual country [3]. As a region under the influence of Confucianism, Taiwan has embedded patriarchal values in families and societies where women are considered inferior to men [4, 5]. In the past, trafficking in women and girls was seldom under the spotlight since society largely tolerated commoditization and exploitation of women and girls. In early days, trafficking in women and girls in Taiwan, e.g. adopted daughters between families in Taiwan in the 1970s and sexually trafficked aboriginal girls in Taiwan in the 1980s, were seriously underestimated since the victims were from minority or disadvantaged groups [6]. These women and girls were the hidden victims of sexual and/or labor exploitation who received little help from the authorities because they were from poor families with little social capital.

However, this problem turned more visible when a new disadvantaged group became the target of exploitation and slavery, namely migrant workers since the 1990s to the present day. From 2005 to 2009, Taiwan was listed as Tier 2 in the Trafficking in Persons Report published by the U.S. Department of State. The Taiwanese government therefore passed the 2009 Human Trafficking Prevention Act (HTPA, Taiwan) in order to show the government’s decisiveness to stop human trafficking cases [7]. Under the legislation and related policy framework, the problem of trafficking domestic women and girls has finally started to catch the attention of law enforcement agencies.

### Migrant workers as victims of HT

With the end of cold war, globalization has affected Taiwan. Globalization has accelerated the economic growth of many developing countries through the creation of an internationally integrated market. Economic expansion has demanded cheaper labor in the competitive global market [8]. Taiwan, having a relatively more developed economy in Asia, started to experience labor shortages, and therefore developed a raised demand for migrant workers [8]. As a result, Taiwan began to import foreign laborers, mainly in agriculture, manufacturing, construction, domestic work, and healthcare. At the end of 2015, the total number of migrant workers has reached 587,940, not including more than 20,000 undocumented workers.

The 2014 Global Slavery Index estimated that 35.8 million people in the world and 23.5 million people (65.8%) in the Asian pacific region were subjected to modern slavery [3]. However, existing reports which include the Asian Pacific countries or territories provide only limited information on HT in Taiwan. First of all, international reports for comparison purposes often emphasize cross-border trafficking and neglect domestic trafficking [7, 9, 10]. Secondly, different reports produced with different methodologies often provide mixed Taiwan cases, either overstatement or understatement. For example, in the 2016 Trafficking in Persons Report by the U.S. Department of State, Taiwan is described as “a destination for men and women subjected to forced labor and sex trafficking and, to a lesser extent, a source of men and women subjected to forced labor and of women and children subjected to sex trafficking” [11]. A similar account is found a decade ago in the 2006 United Nations report which defines Taiwan as one of the major destination regions of HT in Asia [9]. The US report implies that more than 587,000 migrant workers in Taiwan are in a vulnerable situation, increasing the risks of HT, which might be an overstatement. In another report, namely the Global Slavery Index, Taiwan is listed 152 among 167 countries and 25 among 27 Asian Pacific countries by prevalence of population in modern slavery [3]. The Global Slavery Index (2014) estimates the population in Taiwan who lives in the status of modern slavery to be as low as 0.013%. These polarized accounts suggest that the current situation of HT in Taiwan remains an empirical question which is worth exploring.

### The legal definition of HT in Taiwan

The availability of HT data is subjected to the legal definition of HT in Taiwan. According to the HTPA passed in 2009, the legal definition of HT in Taiwan is categorized into four different types: 1) sexual exploitation; 2) labor exploitation with force; 3) labor exploitation without force; and 4) sexual or labor exploitation of minors.

First of all, sexual exploitation is defined as “anyone using such means as debt bondage or another person’s inability, ignorance, or helplessness to force him/her into sexual transactions for profit” (Article 31). The second and third types are both “labor to which pay is not commensurate with the work duty for profit”, with the prior (Article 32 section 1) more serious than the latter (Article 32 section 2). The second type of labor exploitation is punishable by up to 7 years imprisonment and an NT$5 million fine since it involves such means as “force, threat, intimidation, confinement, monitoring, drugs, fraud, hypnosis, or other means against another person’s will”. The third type of labor exploitation is punishable by up to maximum 3 years in prison and a NT$ 1 million fines. The means of labor exploitation involves taking advantage of “another person’s inability, ignorance, or helplessness”. The final type is either sexual or labor exploitation to minors, including acts “to recruit, trade, take into bondage, transport, deliver, receive, harbor, hide, broker … to subject people under 18 years of age to sexual transactions, labor to which pay is not commensurate with the work duty”. In cases of victims under 18 years old, a violation of the HT act holds regardless of whether the work is against the victim’s will or not.

In addition to the HTPA, offenses of HT also include acts conducted in violation of the Criminal Code, Labor Standard Law, or Child and Youth Sexual Exploitation Prevention Act (previously the Child and Youth Sexual Transaction Prevention Act), or other related laws.

## Literature review

### Forms of exploitation

HT might be conducted in various forms in different places, depending on the economic, political, and social context. The pattern and flow of HT might also change over time as a consequence of economic and demographic disparities. In Lee’s literature review of four countries/regions in East Asia, the exploitation forms were divided into sex industry, bride trafficking, bonded labor, illegal adoption, and domestic services [8]. The trafficking of women and girls for sexual exploitation has been the most reported pattern of HT in Asian countries [9]. Sexual exploitation, which appears in the majority of available materials in Japan and Korea, is most frequently cited as a form of HT, with women from Thailand, Philippines, Colombia and Taiwan being trafficked to Japan and South Korea [8, 12]. In recent years, trafficking has become less heard of for Taiwanese women to be trafficked to other countries. Instead, the documented trend is for Chinese women to be trafficked to Taiwan and Hong Kong as sex workers [13, 14].

Forced marriage has been reported in several Asian countries, such as China, both for domestic women and foreign women from Vietnam and North Korea [8]. Forced marriage as an important form of HT in Asian context has been interpreted differently compared to mail-order brides in America or European countries. Forced marriage in Asian context is more akin to commercial marriages described in an Australia report, which found commercial marriages in Australia to be a means of trafficking people for exploitative purposes [15]. However, cross-border marriages through marriage brokers between South-East Asian or Chinese brides for Taiwanese bridegrooms are interpreted differently by Lu, who argues that the women in cross-border marriages in Taiwan act with agency and are not “faceless commodities” [16]. Lu argues that the cross-national marriage phenomenon should be understood in the Eastern context where both paying a bride price and arranged marriages are culturally acceptable and desirable. Whether a woman in cross-border marriage becomes a HT victim depends upon the manifestation of her vulnerability derived from the imbalance of power relations as a result of the nature of matchmaking.

Labor exploitation is also a prevalent form of HT which deserves more attention in the Asian context. Previous studies have identified some vulnerable occupations such as construction, agriculture, restaurants, fishing and catering services, and domestic and care work [10, 17, 18]. Among these risky occupations, migrant domestic servants are more often studied and cited as HT victims. In a qualitative study of Ethiopian domestic workers in Yemen, De Regt describes labor exploitation as commonplace among contract domestic workers for several reasons [19]. First of all, the only legal way to work as a contract domestic worker was to live with the employers which limited Ethiopian women’s mobility and thus contributing to their vulnerability to slavery. Secondly, if a woman chose to work illegally as a freelancer, she was more likely to work under exploitative or even abusive conditions, being denied the right to negotiate salary and workload. Lacking access to legal job offers, Ethiopian women usually became trafficking victims because of the money borrowed from recruitment agents turning into debt bondage [8, 19].

### Perpetrators

The UN trafficking protocol places emphasis on organized trafficking through criminal networks which are dominated by men [20]. The prevailing legal perspective has influenced existing empirical work which focuses on hierarchical and core traffickers, where both are criminal organizations using force and violence to traffic persons for profit [9]. The empirical support for the legal discourse is, however, limited. In Zhang’s study of pimping in a Mexican border city, no evidence was found for systematic collaboration of these pimps with criminal organizations [21]. Among the 92 pimps interviewed, most of them worked alone and had other jobs besides pimping. This coincides Chen’s study in Taiwan, which identified the pimps who transported Chinese women to Taiwan for sex work as underclass males, many of them taxi drivers, who were marginalized by the mainstream economy [13].

As to the nationality and gender of traffickers, previous research shows mixed findings. Some argue that HT is an underground economy which is dominated by male perpetrators. In Zhang’s study of Mexican border city pimps, 83.7% are male [21]. Siegel and de Blank also reported that 81% of the HT suspects registered to the Netherlands in 2004 were men [22]. Other reports, such as cross-national comparison reports, suggested that the traffickers and their victims are homogeneous in terms of gender and race, meaning that trafficked women and girls usually are under the control of women traffickers from the same country of origin [2, 9].

### Victims

Although advocates try to acknowledge that anyone might become a victim of HT, the people who are at higher risk of victimization often come from vulnerable groups, including undocumented immigrants, runaway youths, women and the poor [23]. Fuchs, on the other hand, argues that both legal and illegal migrant workers are at risk of HT in Taiwan [10]. For legal migrant workers, they might be the victims of their own employers or organized illegal brokers, both before or after they become undocumented. This suggests that victimization built into the employment structure might be the root cause of the high volume of migrant workers who become HT victims.

Victims’ demographic characteristics are highly associated with the demands of the labor market, putting women and girls at higher risk of exploitation [24]. Attractive targets of HT also show some quality that makes them potential victims, such as a young age, rural residence, and limited job experience [8, 14]. In the Netherlands, victims of labor exploitation are characteristically undocumented migrants, legal migrants who have limited access to the labor market, women with a dependent residence permit, and marginalized Dutch nationals [17]. The majority of HT victims in the Netherlands are between 18 and 30 years old, younger than their traffickers [22].

Since the definition of HT emphasizes the use of vulnerable situation as a means of exploitation, women and girls under sex trafficking are more likely to be reported in the literature. However, some researchers criticize the traditional image of trafficked women as involuntary, helpless and vulnerable and who are forced into sex work by their traffickers, as not speaking for all women sex workers. For example, Chen’s study on Chinese migrant sex workers in Taiwan concluded that these women did not consider themselves to be exploited victims [13]. McCabe, on the other hand, include those who are initially voluntary but are later placed in unacceptable and undesired working conditions as sexual exploitation victims [24]. McCabe also stresses that the situation of victims who initially consented to the work might be even harsher than those who are forced or deceived into sex work. These victims are usually reluctant to report the crime and when they report, they are usually perceived as unworthy of legal assistance.

Emerton and colleagues go further to deny victim status for people who have been trafficked through “abuse of a position of vulnerability”, questioning whether “vulnerable situation” is a means of exploitation [14]. After interviewing 58 mainland Chinese women in the sex industry in Hong Kong, Emerton and colleagues estimated that only one in five of these women can be identified as HT victims according to the definition of human trafficking in the Protocol to Prevent, Suppress and Punish Trafficking in Persons, Especially Women and Children [14]. Under this conceptual binary, the line between sex trafficking and voluntary sex work is usually hard to draw, causing many victims to be treated as criminals by the criminal justice system in the context where sex work is illegal [14, 25]. Sexually exploited victims often choose to remain in the country they have been trafficked to for sex work [26]. In other cases, the use of force or deception sometimes only appears in the early days of sex trafficking, and the level of violence decreases or disappears when the trafficked victims become more submissive and cooperative, knowing that their choices are limited [26, 27].

The growing number of women migrant workers in Taiwan has created a new risk group of HT, which has become the focus of academic and political endeavors. Given the complicated nature of defining HT, the picture of HT in Taiwan remains an open question. Existing literature in Taiwan and elsewhere have focused mostly on trafficked women and girls from abroad. Native victims of HT are largely disregarded from the picture. Our understanding of HT in Taiwan will not be sufficient without considering both domestic and foreign victims along with the current victim identification under the HTPA. In addition, the forms of women trafficking and the characteristics of victims and traffickers must be investigated under the unique gender, labor, and migration policies and practices of Taiwan.

## Methods

### Research aim

This study attempts to categorize the cases investigated by the law enforcement agencies after Taiwan’s passage of HTPA in 2009. The current HT legislation covers both domestic and foreign victims of trafficking in Taiwan. According to the Legislative Yuan (i.e. the legislature), Taiwan HTPA endorsed the spirit of both Protocol to Prevent, Suppress and Punish Trafficking in Persons, Especially Women and Children, and the US’s TVPA (Trafficked Victims Prevention Act). The aim of this current research is: first, to identify and describe the typologies of reported HT in Taiwan using court proceedings; second, to discuss the characteristics of HT victims, offenders and trafficking process of the detected cases in Taiwan and compare the findings with research literature in other countries.

### Data collection

The main research method of this paper is court proceeding analysis. I searched, with the keyword “Human Trafficking”, 21 district court proceedings during 2009 to 2012. Initially 132 proceedings were identified. The aim was to depict the current situation and result of prosecution cases with women and girls victims of HT. Two conditions must be met: firstly, the offences must be prosecuted as a HT violation and the HTPA must be mentioned; and secondly, cases must involve women or girls as victims. If the cases which meet the above two conditions have male victims, the male victims were excluded from the analysis. In addition, cases were excluded if the proceedings were 1) not related directly to HT (4 cases); 2) offences conducted before the HT Protection Act (36 cases); 3) not prosecuted by the HT Protection Act (40 cases); 4) mistakenly recorded (4 cases); and 5) with no identifiable women or girls as victims (11 cases). As a result, the number of analyzed court proceedings totals 37 cases, involving 195 women and girls victims of HT.

### Data analysis

All of the 37 court proceedings were downloaded from the Judicial Yuan Law and Regulations Retrieving System [28]. These proceedings were firstly carefully reviewed. Secondly, a coding book was developed, comprising 20 variables in four groups. A quantitative dataset comprising 37 cases is created for later analysis.

The first group of variables is the case background information, including court, processing days, procedure, and investigation unit. The second group of variables is violation types which consist of: HT prosecuted, pimping related criminal code violation, violence-related criminal code violation, child and youth sexual exploitation violation, and Labor Standards Act violation. The third group concerns the content of verdict with three variables: verdict, offence type, and exploitation type. The final group is the characteristics of the victims and offenders, including number of victims, number of minor victims, nationality of victims, number of non-citizens, entry methods, illegal status, number of prosecuted suspects, number of HT prosecuted suspects, number of HT guilty offenders, and number of offenders subjected to imprisonment sentencing.

Each case was firstly summarized according to the above 20 variables. They were later recoded as numbers based on the coding book to produce a dataset. Cases are firstly grouped into foreign victims (#1~#21) and domestic victims (#22~#37). Both coding book and dataset are included in Additional file 1.

## Results

The results show that adult sexual exploitation forms the largest share (40.5%) of this sample, followed by adult labor exploitation (27%) and child and youth exploitation (27%). Only one case involves both sexual and labor exploitation where an adult woman was trafficked. More than half of the cases (56.8%) involve adult foreign victims; and 43.2% involve domestic victims (Table 1).

**Table 1:** Case characteristics (*N* = 37)

Factor	Items	Case number	Percent
Exploitation type			
	Adult sexual exploitation	15	40.5
	Adult labor exploitation	10	27
	Adult sexual and labor exploitation	1	2.7
	Child and youth exploitation	10	27
	Not applicable	1	2.7
Nationality of victims			
	Foreign	21	56.8
	Domestic	16	43.2
Prosecuted offences			
	HT and pimp	16	43.2
	HT and violence	9	24.3
	HT and child/youth sexual exploitation	8	21.6
	HT and labor standard act	1	2.7
Verdict cases			
	Not guilty	9	24.3
	Guilty of other offences	10	27
	Guilty of HT offences	18	48.6

The majority of the cases (43.2%) were prosecuted for HT and pimping-related criminal code violations, followed by HT and violent acts (24.3%), HT and child/youth sexual exploitation (21.6%), and HT and the Labor Standard Act violations (2.7%) (Table 1).

As for the verdict outcome, nearly half of the cases (48.6%) were guilty of HT-related offences. Another 10 (27%) cases were prosecuted under other non-HT offences. Nearly one in four cases (24.3%) resulted in not guilty verdicts of any offences. The number of days passed from investigation to court verdict ranges from 145 to 1147, with an average of 485.6 days.

The 37 cases involve a total of 195 women and girl victims. More than half of the victims (53.8%) are foreigners, including migrant workers, marriage immigrants, and those who used a tourist visa to work illegally. Another one in five are minors under the age of 18 (20%) (Table 2).

**Table 2:** Victim characteristics (*N* = 195)

Factor	Items	Persons	percentage
Trafficked women and girls (*N* = 195)			
	Adult citizens	51	26.2
	Adult non-citizens	105	53.8
	Minors	39	20
Sexual exploitation of women and girls (*N* = 107)			
	Immigrant	58	54.2
	Non-immigrant	49	45.8
Labor exploitation (*N* = 46)			
	Immigrant	46	100
	Non-immigrant	0	0
Minor exploitation (*N* = 39)			
	Immigrant	0	0
	Non-immigrant	39	100
foreign women victim entry means (*N* = 105)			
	Marriage immigrant	14	13.3
	Industrial labor	2	1.9
	Caregiver	64	61
	Tourists	15	14.3
	Unknown	10	9.5

Among the victims, 107 women who encountered sexual exploitation and 58 (54.2%) are immigrants, 49 (45.8%) are non-immigrants. In these court cases, labor exploitation only happens to women immigrants, and minor exploitation only happens to domestic girls.

Among the 105 foreign victims, the majority are caregivers who entered as social welfare labor immigrants (61%). They are followed by tourists (14.3%), marriage immigrants (13.3%), and industrial labor (1.9%) (Table 2).

Prosecuted perpetrators of HT in court cases comprised mainly domestic males (*N* = 114) exploiting foreign women (*N* = 105) or native minors (*N* = 39). More than 96% of the offenders are Taiwan natives. Only 35.6% of offenders are found guilty (Table 3). In my study, I have found that the number of prosecuted traffickers in each case ranges from one to 11, with an average of 3.2 persons.

**Table 3:** Offender characteristics (*N* = 118)

Factor	Items	Persons	percentage
Nationalities			
	Domestic	114	96.6
	Foreign (Indonesian)	4	3.4
Verdict outcome			
	Guilty	42	35.6
	less than 6 months	4	3.4
	6 months to less than 1 year	17	14.4
	1 year to less than 2 years	10	8.5
	2 years or more	11	9.3
	Not guilty	75	63.6
	No further action	1	0.8

The 37 cases are categorized into six patterns of HT according to victim characteristics. Firstly, they are divided by the country of origin into native or foreign. Secondly, foreign victims are either legal immigrants (e.g. migrant workers and marriage immigrants) or illegal immigrants according to their entry methods. If legal immigrants who enter via work visa overstay or choose to escape, they might become undocumented when exploitation occurs. For illegal immigrants, the victims are categorized by whether they entered by a fraudulent marriage or other means.

**Fig. 1:**
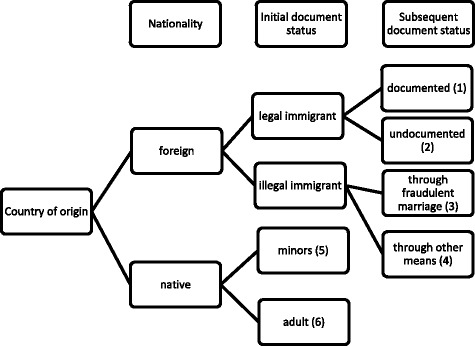
A Typology of Women and Girls HT Victims. Six forms of HT victims are identified according to their country of origin, vulnerability status, and means of transport

In each of the six different types of HT, I will take only one example which resulted in a conviction either of HT or for a violation of the criminal code (Fig. 1).

1. Documented migrant worker under exploitative working conditions (#2,#4,#5,#11,#15,#16,#19,#20)

Before the HTPA was published and enforced, a lot of migrant workers suffered from exploitative working conditions without any attention from the authorities. Since the HTPA defines labor exploitation, contract labor might fall into labor exploitation if it involves 1) using force or other means against one’s will; 2) taking advantage of another person’s inability, ignorance, or helplessness; and 3) as the consequences of the previous two ways, the pay is too low to commensurate with the work. Case #5 shows an example of where employers exploit women migrant workers.

*Case Story #5*

*A couple who ran a nursing home for old people had hired six Vietnamese women workers as caregivers since 2008. The couple sometimes drove the six workers to work as morticians in their funeral service business after regular shifts. The women were asked to work extra hours for less than 2 US dollars per hour. The six women workers were forced to clean and prepare the dead bodies against their will, out of fear for deportation if they refused. It lasted for nearly one year until someone reported the case to the police in September 2009.*

2. At-large migrant workers under exploitation (#1, #3,#6, #8,#12)

At the end of 2015, the statistics showed that 23,149 migrant workers ran away from their workplace, and nearly half (48.4%) were women workers. According to the Trafficking in Persons Report 2016 (Taiwan), this is related to the fact that some migrant workers are subject to debts, usually including: high placement fees from their country of origin, monthly loan payback, and broker service fees in Taiwan. It is not uncommon for migrant workers to “earn significantly less than the minimum wage” after the deduction of the above payments [11]. Those who choose to escape and find jobs on their own in order to earn more money might become vulnerable to HT.

*Case Story #1*

*Eight Taiwanese people and two Indonesian at-large workers housed more than a dozen undocumented Indonesian women workers for sex work. Only 9 victims were found and identified. The case was investigated after police identified one of the victims as at-large immigrant labor. The pimp took nearly 60 percent income of each sex trade from the victims.*

3. Immigrants through fraudulent marriages (#10,#18,#21)

The 2016 Trafficking in Persons Report mentions that women are “lured to Taiwan through fraudulent marriages…for purposes of sex trafficking” [11]. These women, usually from China, are often disadvantaged and vulnerable since they lack other legitimate means to enter Taiwan. They receive less sympathy because they are viewed as criminals who have come to Taiwan voluntarily into sex work.

*Case Story #10*

*A Taiwanese male pimp contacted a Chinese woman and arranged a fraudulent marriage in order to transport the victim to Taiwan during 2009. The above Chinese woman, though willing to be a sex worker, was forced to work under exploitive conditions to pay a debt of around 9,500 US dollars, including flight, fraudulent marriage, and fraudulent husband expenses. Another male Taiwanese driver was responsible for driving the Chinese woman to a motel for sex work. Before the crime was discovered by the police, the Chinese woman only received around 3 US dollars per day. In this case, the man who paid for the expenses of the fraudulent marriage and flight ticket has never been identified. Neither the fraudulent husband nor the driver was prosecuted for HT. The sentence of the male pimp was four years and six months under the HTPA and criminal code.*

4. Trafficked women for sexual or labor exploitation (#7, #9,#13,#14,#17)

In Taiwan, some traffickers would provide fraudulent employment offers to recruit women abroad. These women usually could not afford a legal way to work in Taiwan. Once arriving, the traffickers then use high recruitment fees and debt to force them into sex work. Traffickers will normally use threats and confiscate travel documents to control and detain these women. Although some foreign women know that they will enter sex work before they come, the traffickers often will force them into exploitive working conditions, such as working long hours without vacation and under a debt bondage.

*Case Story #9*

*During 2009, eight Indonesian women were found to have trafficked for sex by a Taiwanese pimp group and an Indonesian pimp group. Four women were lured to Taiwan through deceptive employment offers and another four women through voluntary of sex work. The Indonesian pimp group recruited young women and sent them to Taiwan while the Taiwanese pimp group members then picked them up from the airport and restrained them in a flat. All of the victims were initially forced to have sex with 300 male customers to pay for their travel expenses (5,000 US dollars). One of the victim's relatives reported the case to the Taiwanese police and the Taiwanese pimp members were investigated and prosecuted during 2010.*

5. Underage Taiwanese subjected to sexual exploitation (#26,#27,#28,#29,#30,#31,#33,#34,#35,#36,#37)

The offenders who broker native adult women will usually also broker girls under 18 years old (#30, #33). Typical cases involve both domestic victims and domestic offenders. The offenders are usually club owners, agents, or friends of the girls.

*Case Story #26*

*The major offender ran a night drinking club in Southern Taiwan. In order to bring more business, the major offender recruited young girls under 18 years old as waitresses. Male customers could pay for the girls who drink alcohol with revealing clothing or other sexual services.*

6. Non-immigrant adult sexual exploitation (#22, #23,#24,#25, #30, #32, #33)

The typical non-immigrant adult sexual exploitation contains both Taiwanese offenders and Taiwanese victims. It is also noteworthy that all of the domestic victims are subjected to sexual exploitation, not labor exploitation. Within the seven cases identified, four cases involve call stations which broker sex services by transporting the women to hotels or motels. One case (#32) involved forcing women to sign contract involving disproportionately high penalties which will draw them into vulnerable situation. Only one case (#23) involved trafficking Taiwanese women to Australia for sexual exploitation. None of these cases constituted a violation of the HTPA in court verdict.

*Case Story #32*

*Before the women victim left her hometown and came to Taipei, she was told by a friend that she was offered a chance to begin a career as model. Once she arrived in Taipei, she was forced to sign a contract with the so-called model agent. After that, she was forced into sex work with threats of high penalties for contract violation. After each client, the victim received less than half of the payment.*

## Discussion

### Patterns of reported HT in Taiwan

Prosecuted cases of HT among women and girl victims under the HTPA in Taiwan are dominated by sexual exploitation of women and girls, which aligns with previous literature [1, 2]. Unlike labor exploitation, which is only found among migrant workers in this study, sexual exploitation included both adults/minors and immigrants/natives. According to the analysis, sexual exploitation seems to be the major challenge for Taiwanese authority. However, the issue of sexual exploitation is complicated since sex trafficking in its modern form is not like what Burke described as “a commercial sex act is induced by force, fraud, or coercion” [23]. Instead, the commercial sex act is induced by providing job opportunities in the informal and underground economies for girls, adults, and women immigrants who are struggling to find a decent paying job in the market. This study shows that sexual exploitation in Taiwan is a shared experience for disadvantaged domestic and immigrant women. In addition, minors and illegal immigrants are especially vulnerable to the consequences of sexual exploitation.

More sexual exploitation than labor exploitation is reported to authorities in Taiwan. Subsequently, sex trafficking is more often prosecuted than labor trafficking. This is because commercial sexual transactions through pimps are still considered to be criminal behavior in Taiwan. Law enforcement agencies might put more effort into investigating potential cases of sex trafficking rather than labor trafficking since the chances of proving the incident and achieving a successful prosecution are higher for sex trafficking. In this study, sexual exploitation might have many forms other than pimping and commercial sex work. Sexual exploitation might involve the exploitation of victim’s labor, body, and self, namely “the very personhood of the victim/survivor” [15]. In previous literature, sexually assaulting women also appears as a means of controlling trafficked women both inside and outside of the sex industry [15, 23]. In 2017, the BBC published a case of Indonesian women migrant worker in Taiwan being raped by her boss’s brother [29]. In Cindy Sui’s report, the victim was raped three to five times a week for 1 year, in addition to working more than 16 h per day without any holiday for 16 months. In the end, the prosecutor decided not to file charges and the case fell into legal limbo. This case is believed to be just tip of the iceberg, according to a non-profit organization which supports migrant workers. Since current legislation confines sexual exploitation to sexual transactions for profit (sex work), other forms of sexual exploitation (e.g. pornography, sex slavery, or bride trafficking) might be overlooked by the system. Women’s sexual violence victimization experience might be neglected when they are identified as victims of labor exploitation.

### Perpetrators and the decentralization of HT

Recently, the so-called “decentralization of trafficking” discourse rejects the relationship between organized crime and HT. Perpetrators are believed to be more “free floating”, meaning that the relationship between recruitment, movement and exploitation is much more difficult to prove [30]. This has multiple explanations. First, it might indicate that legal transportation routes are convenient and enough potential victims are available from legal routes. Therefore, there is no need to include multiple traffickers in the HT process. Secondly, the number of prosecuted offenders is small, probably because of the difficulties of tracing and making connections with the offenders who initially recruit and transport persons since many offenders hide their identities.

Since the Taiwanese government abolished commercial matchmakers in August 2009, trafficking women through fraudulent marriages has decreased. Instead, individual perpetrators who induce foreign women through the Internet or social media have become more and more frequent. Different from European and many other countries where perpetrators and victims usually come from the same country of origin [2, 9], the Taiwanese legal cases show that Taiwanese men were charged in cross-border trafficking of women. These legal case data shows little evidence that Taiwanese women were charged as recruiters, entrepreneurs, or managers in HT. Many of the perpetrators work alone, involving no criminal organizations.

### Victims, victim identification and stigma

This current research confirms that domestic trafficking is at least as much reported as cross-national trafficking, mostly involving trafficking of girls. Nevertheless, the legal case descriptions in this study show that the court decisions indicate that foreign victims and minor victims are more likely to be viewed as “real” victims of HT. The content of the verdict also shows that some victims are stigmatized as voluntary sex workers (#7, #22, #23, #25). In fact, the police are often informed of these incidents through a third person, such as their friends or relatives. Domestic victims might be more concerned about being blamed for causing their own victimization, and they are thus reluctant to disclose their experiences to law enforcement [31]. In other studies, native adults were considered incapable of being victimized, e.g. Cottingham et al. describes “willing participants who have asked for it” [30]. The criminalization of sex work also might deter victims’ willingness to seek help [32].

More than one in four cases do not result in convictions for HT offenses, indicating that HT is likely to be misidentified as other types of offences. Victim identification is even more complicated when the perpetrators are employers or intimate partners. Judges tend to categorize these cases as civil disputes or violent incidents rather than HT. Similar to the victims of marriage trafficking, perpetrators’ controlling behaviors, such as physical or sexual violence, are likely to be misidentified as domestic violence. Identifying HT victims remains a challenge for both law enforcement and social welfare agencies in Taiwan. Even when the behaviors are criminalized and punished by law, the identification of victims is often accompanied by the blaming of the victims.

### Causes of victimization

Cottingham and her colleagues point out that, in a global economy, low wage work is fulfilled by individuals from developing nations or native born people in wealthy nations with limited education and skills [30]. My study is in line with the Trafficking in Persons 2016 Report suggestion that foreign caregivers, constituting 61% of the victims in my sample, are an especially vulnerable group [11]. Taiwan has become a destination of HT since Taiwanese society is in short supply of women laborers of low-wage work. With increasing gender equality, the employment rate of women population has increased from 33.3% in 1980 to 55.9% in 2013. The increasing market for domestic servants and child/elder care creates a need for migrant women workers [33]. These workers, either as an integral part of the formal economy or part of the informal economy (undocumented, untaxed and unorganized workers), are usually vulnerable to exploitation. In a society with higher levels of inequality, the potential for exploitation increases [3].

Gender and ethnicity are two of the reasons some people are isolated and marginalized in a society, which makes women more likely to be adversely affected by the social and economic factors in their country of origin [34]. Women and girls are often discriminated against and marginalized, and excluded from education and employment opportunities in their countries of origin [8, 35]. Like underage girls who have been sold and enslaved in Taiwanese history, the vulnerability of women migrant workers is because of the tolerance of legal exploitation by registered recruitment services. It also applies to the native women victims who encountered sexual exploitation. The research findings suggest that the vulnerabilities of trafficked women and girls might be better understood if the factors of their disadvantages in the local employment market are included.

### Limitations

This study was conducted through a small number of cases obtained from the Judicial Yuan Law and Regulations Retrieving System. Like many other countries in the world, very few human trafficking cases were brought into court in Taiwan. This study also excluded some trafficking like cases which were not prosecuted under the HTPA. The dataset may not represent the whole picture of women trafficking in Taiwan as many victims are hidden. A lot of the literature points to the complexities and difficulties in identifying victims of HT [34]. More empirical studies in Asian contexts will contribute to a better understanding of this pressing issue for women’s welfare.

## Conclusions

This study is the first attempt to quantify the court verdicts of HT cases in order to get a glimpse of the characteristics of victims, offenders and situations in an Asian society. This study intends to improve our understanding of HT in a local context in Taiwan. Using the court proceedings from 2009 to 2012, six forms of HT are identified based on 37 HT cases, involving 195 women and girls victims and 118 offenders. The six types of HT cover both domestic and cross-border trafficking, which affect both immigrants and citizens. This analysis also shows that labor trafficking was only reported among migrant workers, which indicates that their vulnerability to exploitation might be rooted in the employment structure.

The Global Slavery Index report has pointed out the relationship between discrimination and level of slavery [3]. Lee also proposes that the wider social mechanism forms the “push” and “pull” factors underlying the vulnerability of women and children in East Asia [3]. From a macro-level sociological perspective, gender inequality and gender-based violence in families and societies contributes to the feminization of migration [35]. De Regt therefore argues that gendered migration means “increased mobility” for those women “who were previously confined to their home, villages, or neighborhoods” [19]. In that sense, HT victims in Taiwan, namely young women and girls from Taiwan or abroad, have consciously chosen to escape from one high risk situation (e.g. their homes or hometowns) to another (e.g. big cities or other countries) to avoid victimization by the social and/or economic system. Drawing from Routine Activity Approach by Cohen and Felson [36], the modern social structure creates more opportunities for activities such as trafficking young women and girls because the chances of convergence between potential victims and motivated traffickers have increased by urbanization, internet, and the dispersion of activities away from households and families. In that sense, young women and girls’ victimization has more to do with the risks in routine activities, rather than systematic recruitment and transporting by organized crime groups [37].

What is different for women migrant workers, however, is their non-citizen status. The status of non-citizenship will keep them from serious criminal justice system attention because of lack of political power such as the rights to vote, causing the trafficking to remain underground [38]. In order to raise the awareness of HT, we need to address the economic and social factors that give rise to HT globally and locally.

## Additional file

Additional file 1: This file consists of coding book and tabular data of all 37 cases analyzed in this paper. (XLSX 14 kb)

## Abbreviations

HT: Human trafficking
HTPA: Human Trafficking Prevention Act
TVPA: Trafficked Victims Prevention Act

## References

[1] Shelley L, Bergoffen D, Gilbert PR, Harvey T, McNeely CL (2011). Human trafficking: why is it such an important women's issue?. Confronting global gender justice: women's lives, human rights.

[2] UNODC (2016). Global report on trafficking in persons 2016.

[3] Hope for Children Organization Australia Ltd (2014). The global slavery index 2014.

[4] Liu C-L (2014). Mental health problems in Taiwan from a gender and anti-oppressive perspective. Socialno Delo.

[5] Xu X, Lai S-C (2002). Resources, gender ideologies, and marital power: the case of Taiwan. J Fam Issues.

[6] Wu DYH, Korbin JE (1981). Child abuse in Taiwan. Child abuse and neglect: cross-cultural perspectives.

[7] Chen Y, Hofmeiser W, Rueppel P (2014). Human trafficking in Taiwan. Trafficking in human beings: learning from Asian and European experiences.

[8] Lee JJ (2005). Human trafficking in East Asia: current trends, data collection, and knowledge gaps. Int Migr.

[9] Fowke M, Aronowitz AA, Sarrica F, Albert S, Symalzek J (2006). Trafficking in persons: global patterns.

[10] Fuchs R (2011). Human trafficking of legal and illegal migrant workers in Taiwan.

[11] US Department of State (2016). Trafficking in persons report Taiwan 2016.

[12] Jones L, Engstrom D, Hilliard P, Sungakawan D (2011). Human trafficking between Thailand and Japan: lessons in recruitment, transit and control. Int J Soc Welf.

[13] Chen MH (2011). Exploitation or reciprocity? The intersectionality of gender, class and sexuality in cross-strait commercial sex networks. Tawanese J Sociol.

[14] Emertons R, Laidler KJ, Petersen CJ (2007). Trafficking of mainland Chinese women into Hong Kong's sex industry: problems of identification and response. Asia Pac J Hum Rights Law.

[15] Lyneham S, Richards K (2014). Human trafficking involving marriage and partner migration to Australia.

[16] Lu MC-W (2005). Commercially arranged marriage migration: case studies of cross-boarder marriages in Taiwan. Ind J Gend Stud.

[17] Smit M (2011). Trafficking in human beings for labour exploitation: the case of the Netherlands. Trends Organized Crime.

[18] McKinnel T, Lee JYC, Salmon D (2016). Made in Taiwan: government failure and illegal, abusive and criminal fisheries.

[19] de Regt M (2010). Gender, mobility, and il/legality among Ethiopian domestic workers in Yemen. Gend Soc.

[20] Breuil BCO, Siegel D, van Reenen P, Beijer A, Roos L (2011). Human trafficking revisited: legal, enforcement and ethnographic narratives on sex trafficking to Western Europe. Trends Organized Crime.

[21] Zhang SX (2011). Woman pullers: pimping and sex trafficking in a Mexican border city. Crime Law Soc Change.

[22] Siegel D, de Blank S (2010). Women who traffic women: the role of women in human trafficking networks - Dutch cases. Glob Crime..

[23] Burke MC, Burke MC (2013). Introduction to human trafficking: definitions and prevalence. Human trafficking: interdisciplinary perspectives.

[24] McCabe KA, Burke MC (2013). Common forms: sex trafficking. Human trafficking: interdisciplinary perspectives.

[25] Finn MA, Muftic LR, Marsh EI (2015). Exploring the overlap between victimization and offending among women in sex work. Vict Offenders.

[26] Demir OO (2010). Methods of sex trafficking: findings of a case study in Turkey. Glob Crime.

[27] Hopper E, Hidalgo J (2006). Invisible chains: psychological coercion of human trafficking victims. Intercultural Hum Rts L Rev.

[28] Judicial Yuan law and regulations retrieving system. http://jirs.judicial.gov.tw/Index.htm. Accessed 28 June 2017.

[29] Sui C (2017). Taiwan’s shameful secret [BBC news].

[30] Cottingham M, Nowak T, Snyder K, Swauger M, Burke MC (2013). Sociological perspective: underlying causes. Human trafficking: interdisciplinary perspectives.

[31] Menaker TA, Franklin CA (2015). Gendered violence and victim blame: subject perceptions of blame and the appropriateness of services for survivors of domestic sex trafficking, sexual assault, and intimate partner violence. J Crime Justice.

[32] Armstrong L (2017). From law enforcement to protection? Interactions between sex workers and polic in a decriminalized street-based sex industry. Br J Criminol.

[33] Lan P-C (2006). Global Cinderellas: migrant domestics and newly rich employers in Taiwan.

[34] Lange A (2011). Research note: challenges of identifying female human trafficking victims using a national 1-800 call center. Trends Organized Crime.

[35] Siantz MLL (2013). Feminization of migration: a global health challenge. Glob Adv Health Med.

[36] Cohen LE, Felson M (1979). Social change and crime rate trends: a routine activity approach. Am Sociol Rev.

[37] Kenyon SD, Schanz YY (2014). Sex trafficking: examining links to prostituion and the routine activity theory. Int J Criminol Sociol.

[38] Weber L (2013). Policing non-citizens.

